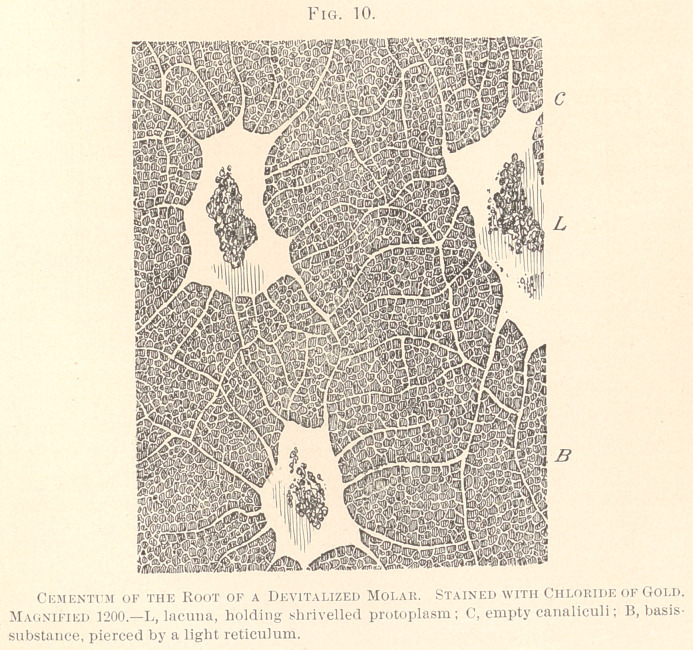# A Contribution to the Minute Anatomy of the Cementum

**Published:** 1892-10

**Authors:** C. Heitzmann, F. A. Roy


### International Dental Journal.

Vol. XIII. October, 1892. No. 10.

### Original Communications.[1]

### A CONTRIBUTION TO THE MINUTE ANATOMY OF THE CEMENTUM.[2]

BY C. HEITZMANN, M.D., AND F. A. ROY, M.D., D.D.S.

#### HISTORY.

C. F. W. Bödecker, in his essay, "The Distribution of Living Matter in Human Dentine, Cement, and Enamel" (*Dental Cosmos*, 1878–79), says of the cementum, "Delicate parallel striations are to be seen in the cementum, identical with the lamellæ of an Haversian system, and, as a rule, more plainly marked near the periphery than towards the dentine. Within the basis-substance of the cementum there are numerous branching spaces, in correspondence with the lacunæ of bone. The offshoots of these spaces, like the spaces themselves, are very marked in dry specimens, because of their being filled with air. In chromic-acid specimens, on the contrary, the offshoots are much less pronounced; and the less the more thoroughly the decalcification has been effected by the acid. No essential difference is noticeable between the lacunæ and canaliculi of ordinary bone and those of the cementum;

[1] The editor and publishers are not responsible for the views of authors of papers published in this department, nor for any claim to novelty, or otherwise, that may be made by them. No papers will be received for this department that have appeared in any other journal published in the country.

[2] Read before the New York Odontological Society at their meeting on June 21, 1892.

in both tissues there exists a great variety as to the general arrangement, the size of the lacunæ, and the number and ramifications of their offshoots. The walls of the lacunæ and the coarser offshoots, if viewed with a highly-magnifying lens, appear interrupted at their peripheries by light spaces, which lead into a light, delicate net-work, piercing the whole basis-substance to such an extent that only the meshes have to be considered as the fields of calcified glue-yielding basis-substance. Each lacuna contains a protoplasmic body, with a central nucleus,—the cement-corpuscle. The net structure of the protoplasm is plainly visible in all cement-corpuscles. From their periphery conical offshoots arise, the coarser of which penetrate into the larger offshoots of the lacunæ, while the finest offshoots traverse the light rim between the wall of the lacuna and the periphery of the protoplasmic body, being directed towards the light interruption on the boundary of the lacuna. Numerous cement-corpuscles send broad and branching offshoots through the basis-substance in a vertical or oblique direction to the lamellæ, and not infrequently a direct union is established between two or three cement-corpuscles by means of such large offshoots. In some teeth broad, spindle-shaped spaces pierce the cementum in a radiated direction, all of which contain protoplasm with delicate offshoots directed towards the net-work in the basis-substance. All protoplasmic bodies within the cementum, though greatly varying in shape, agree in being connected with each other by the delicate net-work which pierces the basis-substance. The connection between dentine and cementum is established either by a gradual change of one tissue into the other, without a distinct line of demarcation, or there exists a boundary formed by a more or less marked wavy line, presenting irregular bay-like excavations. Lastly, it occurs that between the bay-like excavations and the dentine there is interposed a stratum of the structure of cementum, with a gradual change of the tissue of the former into that of the latter. Where a boundary with bay-like excavations is present between dentine and cementum, spindle-shaped enlargements of the dentinal canaliculi may be seen. The majority of the dentinal canaliculi, however, reach the boundary of the cementum after repeated bifurcations, by which both the calibres of the canaliculi and their central fibres are gradually diminished in size. A connection of the dentinal fibres with the coarser offshoots of the cement-corpuscles is often observed. The light net-work of the basis-substance of the dentine always passes into that of the cementum."

Of the structure of the cementum at the neck of the tooth this author says, "In the great majority of teeth neither the canaliculi nor their contents, the dentinal fibres, reach that part of the cementum which surrounds the neck, in accordance with the statement first made by John Tomes. Near the periphery of the dentine, bifurcations of the canaliculi, and consequently also of their tenants, the dentinal fibres, take place, some of the finest terminations of which run to the boundary between the dentine and cementum. As a rule, the finest terminations of these fibres are lost to sight in a net-work somewhat coarser than that of the basis-substance of ordinary dentine. Sometimes the dentinal canaliculi, when approaching the periphery, become slightly dilated, so as to produce slender, pear-shaped cavities, in accordance with which the terminating dentinal fibres exhibit slight enlargements. The cementum around the neck forms a narrow layer, cut off obliquely at the place of junction with the enamel. It is built up by delicate prisms or spindles, arranged vertically to the surface of the dentine. The prisms represent the fields of the basis-substance, and are separated from each other by light rims, holding beaded fibres, or traversed by delicate vertical threads. The cementum on the neck of the tooth is devoid of lamellæ and lacunæ, which appear deeper below, together with all the characteristic features of a fully developed structure of the cementum. The lamellæ become the more distinct, and the lacunæ with their contents—the cement-corpuscles—the more numerous, the broader the diameter of the layer of the cementum."

In the conclusion of his essay, Bödecker makes the following statements: "The cementum, as well as ordinary bone, is provided with lacunæ and canaliculi. The lacunæ contain nucleated protoplasmic bodies, and the canaliculi hold offshoots of the living matter of protoplasm. The whole basis-substance of the cementum is traversed by a delicate net-work, which, in all probability, contains living matter, though this is traceable only in its thorn-like projections from the periphery of the protoplasmic bodies and their larger offshoots. The living matter of the cementum is uninterruptedly connected with that of the pericementum, and continuous with the living matter of the dentine, either through intervening protoplasmic bodies in the interzonal layer, or directly with the dentinal fibres. The cementum covering the neck of the tooth is devoid of lamellæ and protoplasmic bodies. It is built up by directly ossified osteoblasts of the pericementum presenting their prismatic shapes, and everywhere traversed by a net-work of living matter. This is in connection with the pericementum and with the dentine mainly through the intervening net-work in the basis-substance of the latter."

The first author who confirmed and accepted Bödecker's views as to the minute structure of the cementum was Frank Abbott. In his paper, "Microscopical Studies upon the Absorption of the Roots of Temporary Teeth" (*Independent Practitioner*, 1884), he claims that the bay-like excavations observed in the cementum and the dentine are due to a dissolution first of the lime-salts, and afterwards of the basis-substance, which causes the reappearance of protoplasm in certain pre-formed territories. The new infiltration of these territories with basis-substance often results, even in the dentine, in a formation of bone-tissue.

Bödecker's claim that all the three hard tissues of the teeth—the dentine, the cementum, and the enamel—are alive in a living organism, although largely based upon assumptions, was fully verified by later observers. Frank Abbott, in 1880[1] and in 1885,[2] directly proved the presence of a delicate reticulum in the enamel, after complete decalcification by means of chromic and acetic acids. John I. Hart, in 1891,[3] has conclusively shown the presence of a delicate reticulum interconnecting the dentinal fibres and pervading the whole basis-substance, by means of a protracted stain with solution of chloride of gold, and subsequent decalcification with acetic acid. The proof of the existence of such a reticulum in the cementum was, however, lacking. In some specimens prepared in C. Heitzmann's laboratory by Dr. William Carr, in 1889, this structure was plainly seen, and last winter we decided to work up this topic more thoroughly than had been done in previous years.

### II. METHOD.

A recommendable method for the study of the microscopical structure of the cementum is to grind a tooth as thin as possible, always keeping it moist with a one-per-cent. solution of table-salt. First a coarse slab is made of the freshly-extracted tooth with a fine saw or with a corundum stone in the lathe, which afterwards is ground thin on a corundum slab, and finished on an Arkansas stone. The specimen is not allowed to become dry for a moment. After brushing away the débris from grinding, the specimen is

---

1 "The Minute Anatomy of Dentine and Enamel" (*Dental Cosmos*, 1880).

2 "The Minute Structure of Enamel" (*Dental Cosmos*, 1885).

3 "Minute Structure of Dentine" (*Dental Cosmos*, 1891).

directly mounted in chemically-pure glycerin, or staining with ammoniacal carmine may be resorted to, though such a stain is of comparatively little value for the study of cementum. Some of our illustrations are made from specimens prepared in the just described manner. They are especially plain when examined immediately after mounting, but rarely fit for higher powers of the microscope, exceeding five hundred to six hundred diameters. After several weeks, when the glycerin has thoroughly saturated the specimen, the minute features are, as a rule, less conspicuous than on the freshly-mounted specimen.

The method first employed by William Carr, in 1889, for bringing to view the minutest structure of the dentine, is a protracted stain with one-half of one-per-cent. solution of chloride of gold of plates ground thin with the finger on grinding-stones and corundum slabs, with subsequent complete decalcification by means of a six-per-cent. solution of glacial acetic acid. This method has also enabled John I. Hart to obtain perfect specimens of dentine. The same procedure we have adopted for clearing up the minutest features in the structure of the cementum. Freshly-extracted teeth are placed in a one-per-cent. solution of table-salt and ground thin, as before stated. It is important to leave the extracted teeth but a short time in the salt-solution,—a few hours at the utmost,—since the protoplasmic formations of the cementum become hydropic and unstainable with chloride of gold if left in the solution for days. This tissue, being directly exposed to the salt-solution, is affected by it sooner than the dentine. Special care must be taken in keeping the slabs moist with a weak solution of table-salt, lest the specimen become dry, and the lacunæ and the coarser canaliculi filled with air, by which the details are rendered indistinct or are completely lost. After the slab—immaterial whether a longitudinal or a transverse section—has attained a sufficient degree of thinness, as proven by mounting on a slide and covering with the thinnest possible covering glass, it must be washed carefully and repeatedly with a camel's-hair brush, under continuous renewal of the salt-solution. Next the slab is placed in a one-half of one-per-cent. solution of chloride of gold, whereby all metallic instruments must be avoided, the simplest spatula being a match shaped with a penknife. If several sections should be ready for treatment with chloride of gold, it is necessary to note that each one be in free contact with the gold-solution. Specimens overlapping one another prevent a perfect stain, because they will not allow the desired contact and penetration with the gold-solution.

The time required for exposure to the gold-salt is somewhat different with different teeth, probably due to the variable amount of lime-salts infiltrating the cementum. The best results we have obtained were on temporary teeth left in the gold-solution from six to seven hours,—certainly a shorter time than is required for staining the dentine. The following exposure for ten hours to a six-per-cent. solution of glacial acetic acid is, as a rule, sufficient for complete decalcification of the cementum, and now the specimen may be exposed to broad daylight for a number of days, until it has assumed a dark-violet or dark-purple color. The only precaution required at this stage of the procedure is to renew the distilled water in which the specimen lies every day, in order to prevent the growth of mildew. A drop of chemically-pure glycerin added to the distilled water is a sure means of keeping away the mildew. The mounting of such specimens is invariably done in glycerin,—the only medium which allows the examination with the highest powers of the microscope.

We admit that the results obtained in the described manner were not uniformly satisfactory, in contradistinction to dentine, in which failures are exceptional. We cannot account for the fact that specimens of different teeth, of which we knew that they were alive and perfectly fresh, yielded good images only in a fractional number. Cohnheim, the discoverer of the gold-stain, met with the same accidents. Sometimes the basis-substance assumes a deeper color than the contents of the lacunæ; the latter may remain even so pale as to imitate features of devitalized teeth. Specimens, however, well stained, exhibited all features so handsomely that we thought best to describe this method, leaving the invention of new procedures to other observers. It is especially the acetic acid which we suspect of yielding unsatisfactory results, and other acids—perhaps diluted sulphuric acid—will have to be resorted to in future trials. Owing to the unequal efficiency of the method employed, we will abstain from statements which are of the utmost importance to the practitioner,—viz., the definition of the living from the non-living part of the cementum in pulpless teeth. The results of our examination seem to be sufficiently interesting, however, as far as they go, to deserve presentation.

### III. CEMENT OF THE ROOTS.

The reason why histologists made so little progress in the recognition of the minute anatomy of bone-tissue generally, was that dry bone was used for study exclusively.

Strange as it may sound, the majority of histologists of all nations still resort to this irrational method, even in our day. As soon as the bone is allowed to dry, all soft parts of the tissue, the seat of life, must perish by shrinkage, and only the frame-work of the basis-substance will be left. This latter is known to be glue-yielding and thoroughly infiltrated with lime-salts. In a dry specimen only spaces, the so-called lacunæ and their offshoots, the so-called canaliculi, could be seen, and the contents of these spaces were thought by some to be a liquid, by others carbonic-acid gas. Charles S. Tomes, in his "Manual of Dental Anatomy, 1876," frankly admits that nothing positive is known as to the contents of the lacunæ. It was twenty-two years ago,—in 1870,—that in Stricker's laboratory fresh, living bone, the thinnest plates of the ethmoid bone of rabbits, were examined microscopically, being kept alive in blood-serum, or in a one-half of a one-per-cent. solution of table-salt. The fact was thus settled that each lacuna holds a nucleated protoplasmic body, the bone-corpuscle proper, from which numerous offshoots arise, penetrating the coarser canaliculi. Not all the canaliculi could be seen to hold protoplasmic offshoots, and even at present the opinion of the best French histologist, L. Ranvier, is that only a limited number of canaliculi contain protoplasmic processus of the bone corpuscles. Obviously a dry bone-specimen, mounted in Canada balsam, showed the lacunæ and the canaliculi filled with air or dirt, and appeared under the microscope exactly like dead or necrotic bone. Still, who will doubt that bone-tissue is alive in a living organism all through, reacts by inflammation upon injuries, grows and is nourished, while a piece of necrotic bone is a foreign body to the organism, and nature makes hard efforts to have it cast off by suppuration of the surrounding tissues.

C. F. W. Bödecker was the first to point out the difference between a living and a necrotic lower jaw-bone under the microscope. In his paper "Necrosis," published in 1878 in the *Dental Cosmos*, he, after softening the bone-tissue with a solution of chromic acid, demonstrated the presence of a protoplasmic body in every lacuna in living bone-tissue; whereas the canaliculi showed prolongations of the protoplasm only in the immediate vicinity of the latter. Dead, necrotic bone, on the contrary, treated in the same manner for decalcification, invariably contained nothing but empty lacunæ, with some scanty, granular remnants of the protoplasm or clusters of micrococci. In both instances the basis-substance appeared to be pervaded by an extremely delicate light filigree, in connection with the lacunæ and the canaliculi.

Since the cementum covering the roots of the teeth is in all its essential features bone-tissue, being lamellated and supplied with a number of radiating lacunæ, the examination of a dry specimen ground thin furnished features identical with those of dry bone. The lacunæ appeared black from being filled with air or dirt, the same as the coarser canaliculi yielding the image of a spider or a crab. If live cementum, immediately after the extraction of a live tooth, be placed into a solution of chromic acid, thin sections could be made with the razor, which, as Bödecker first described, plainly demonstrated the presence of a nucleated protoplasmic body in every lacuna, and offshoots emanating therefrom, which fill the coarser canaliculi; nay, connect with the dentinal fibres at the interzonal layer between cementum and dentine. It was a mere inference that even the finest canaliculi—nay, the most delicate reticulum in the basis-substance—contained fibrillæ of living matter.

We have examined quite a number of roots of teeth, both deciduous and permanent, in specimens ground thin under the protection of an indifferent liquid, a one-per-cent. solution of table-salt. Our intention has been to study the features of the cement-tissue in freshly-ground specimens, before they were exposed to the influence of a solution of chloride of gold. What we saw in many instances has not as yet been described. (See Fig. 1.)

We find, with low powers of the microscope, the surface of the cementum provided with shallow pits, rendering the border line, in a longitudinal section, hilly or wavy. This corresponds to the rough, slightly pitted aspect of the surface of the roots, if viewed with the naked eye. The lamellated portion of the cement gradually tapers towards the neck of the tooth, and becomes the broader, the more it approaches the apices of the roots. In this portion of the cement-tissue, the parallel lamellæ are conspicuous, and we notice a number of cement-corpuscles scattered in the basis-substance, which differ from ordinary bone-corpuscles only by being more irregular, often elongated, apparently arranged without regularity, certainly without parallelism to the lamellæ. Aside from the cement-corpuscles, we notice a number of lines running from the surface to the dentine, and piercing the lamellæ at right or acute angles, often in union with the cement-corpuscles. The course taken by these lines, which somewhat approach in their parallelism the aspect of dentinal fibres, is always devious to the latter, with which they produce obtuse angles. We have failed to find an immediate union of dentinal fibres with the fibres piercing the cementum. The lamellated portion is followed by a layer, indistinctly lamellated or striated, likewise containing cement-corpuscles and likewise traversed by radiating lines. This layer, either indistinctly lamellated or entirely destitute of lamellæ, we propose to term "the osteoid layer of the cementum," and will devote to its description the next chapter of our paper. At the boundary of the osteoid layer, towards the dentine, a layer of numerous small protoplasmic bodies is seen in our specimen similar to those of an ill-calcified enamel, and probably due to a deficient calcification of the cementum. This layer is rather exceptional. The dentinal canaliculi either stop short of the interzonal osteoid layer, or a limited number of canaliculi is seen to directly run into the interzonal layer where they inosculate with the coarse protoplasmic reticulum.

The aspect of the cement-corpuscles in the distinctly lamellated portion is rather peculiar in ground specimens, mounted in glycerin, when viewed with medium powers of the microscope, five hundred to six hundred diameters. The lacunæ appear filled with protoplasmic bodies, in the centre of which we notice oblong nuclei. From the periphery of the bodies numerous offshoots break forth, which either directly connect neighboring cement-corpuscles, or are lost in the basis-substance. Innumerable fine offshoots are seen to emanate from the coarse ones, and an indistinct net-work is established by these finest offshoots, particularly plain in the vicinity of the cement-corpuscles. The basis substance appears coarsely granular, but no reticulum proper can be made out with this amplification. The basis-substance is pierced by peculiar fibres which intersect the lamellæ at right or acute angles, often bifurcate, and in many instances are independent of the cement-corpuscles or their offshoots, while in other instances they distinctly inosculate with the two last-named formations. (See Fig. 2.)

The question arises, What are these fibres in the cementum? The first idea suggesting itself is that we have to deal with perforating fibres, first described by Sharpey in specimens of dry bone-tissue. Charles S. Tomes[1] says, "Like bone, cementum is also sometimes found to contain Sharpey's fibres; that is to say, rods running through it at right angles to its own lamination, and, as it were, perforating it. These are probably calcified bundles of connective tissue."

Sharpey's fibres are never seen in specimens decalcified by solu-

---

[1] "A Manual of Dental Anatomy," Philadelphia, 1876.

tions of chromic acid. Histologists assert that they are elastic fibres, not perishable after treatment of the specimen with caustic potash. The fibres in the cementum, on the contrary, are canaliculi in the basis-substance, holding protoplasm, or rather living matter, which feature renders them akin to dental fibres. We propose the name of "piercing cement-fibrillæ" for their designation.

The ultimate analysis of the tissue of cementum is possible only after exposure to a solution of chloride of gold and subsequent decalcification with acetic acid. (See Fig. 3.)

We see the cement-corpuscles, the nuclei of which often appear a trifle lighter than the rest of the protoplasm. The reticular structure of the latter is plain in many corpuscles. Numerous coarse and innumerable fine offshoots break forth from the periphery of the cement-corpuscles. Close around the latter and their coarse offshoots a light rim is present, traversed by the minutest conical offshoots, which rim obviously corresponds to the wall of the lacunæ and canaliculi. The walls are rendered indistinct by the decalcification of the specimen. The whole basis-substance is traversed by an extremely minute reticulum of a dark-violet color, interconnecting all cement-corpuscles. The behavior of this reticulum towards chloride of gold clears up the nature of it: it is the living matter that produces a net-work in the protoplasm as well as in the basis-substance. The meshes of this reticulum, on the contrary, must be considered as the basis-substance proper, composed of what we term glue or gelatine, and saturated with lime-salts. The boundary zone or interzonal layer between dentine and cementum is conspicuous by its bay-like excavations. In this region we easily recognize the union of most of the dentinal fibres with coarser offshoots of the cement-corpuscles, a fact already established by Bödecker. An immediate union of the living matter of the dentine with that of the cementum is fairly plain, since six to eight hours' exposure to the solution of chloride of gold was sufficient to bring to view the reticulum in the cementum, whereas ten hours' exposure is needed, according to John I. Hart, to stain the reticulum of the dentine to a satisfactory degree. No doubt the whole cementum is a tissue, endowed with properties of life in a living tooth, the same as in dentine. Life is not confined to the cement-corpuscles and their coarser offshoots, but extends all through the basis-substance, being attached to the reticulum that pervades the protoplasm, as well as its product, the basis-substance. The study of pathology of cementum has furnished ample proof for the corroboration of this statement.

### IV. OSTEOID CEMENTUM.

The tissue of the cementum covering the neck of the tooth is different in its microscopical structure from the main mass of the cementum. In the next chapter we propose to dwell more fully on the cementum of the neck. It is known that the thin layer of this tissue gradually blends with the cementum proper. First the prisms of the neck-layer disappear, and a granular layer makes its appearance, destitute, as yet, of lamellæ and cement-corpuscles, exhibiting a few faint striations, and, as in our specimen, though not constantly, traversed by piercing cement fibrillæ. (See Fig. 4.)

This formation is probably what Charles S. Tomes[1] describes in the following words: "The matrix of the cementum is sometimes apparently structureless, at others finely granular or interspersed with small globules." We wish to term it the "osteoid layer" of the cementum. By the name "osteoid" the histologists designate a tissue kindred to bone, saturated with lime-salts, lacking bone-corpuscles or showing but few of them, and without lamellæ. All these properties hold good for the portion of the cementum under consideration. Its history of development will be cleared up some day, when the development of cementum will be studied in human teeth,—something not as yet accomplished.

The osteoid layer shows, upon approaching the lamellated cementum, a few irregular bone-corpuscles. The lamellated portion starts from the periphery of the root, and soon becomes broader and supplied with regular, freely branching cement-corpuscles, whereby the osteoid structure, in the majority of the teeth examined, is lost to sight. In the specimen from which the illustration is taken, the dentinal fibrillæ stop short of the osteoid layer, a feature so characteristic for the anatomy of the neck. Near the interzonal layer, between the dentine and the osteoid tissue, a few small protoplasmic bodies are seen, into which dentinal fibres inosculate.

Not infrequently the osteoid layer does not perish, even though lamellated cementum has made its appearance; nay, the osteoid layer may be seen all around the root, as an intervening formation between dentine and cementum. Figure 5 is taken from such a specimen. Here we see the beginning of a lamellated formation, between which and the dentine are protoplasmic bodies, with a number of long and parallel offshoots, of the aspect of piercing

---

[1] "A Manual of Dental Anatomy," Philadelphia, 1876.

fibres. The interzonal layer, in this instance, is conspicuous by a large number of interconnecting protoplasmic bodies, upward in union with cement-corpuscles, and downward with dentinal fibres. This peculiar formation was traceable all along the roots, and means an incomplete deposition of lime-salts at the time of the formation of the cementum. The dentine of this tooth is fully developed, lacking even the so-called interglobular spaces in the crown.

In order to more thoroughly understand the structure of the osteoid layer, we have applied to such specimens the protracted gold-stain, with subsequent decalcification by means of diluted acetic acid; the image thus obtained was a striking one indeed. (See Fig. 6.)

We observe small, odd-shaped protoplasmic bodies of dark violet color, exhibiting but a limited number of coarse offshoots. Besides, coarse dark-violet fibres traverse the basis-substance which produce a coarse net-work, arranged without an apparent regularity, and, as it seems, also lacking connections with larger protoplasmic masses. This latter feature may be due to a curved course of the fibres, the union with cement-corpuscles being severed by the process of grinding. The whole basis-substance is seen to be traversed by a, comparatively speaking, coarse, dark-violet reticulum, in union with the protoplasmic bodies, as well as with the coarse fibres. Unquestionably the reticulum is the living matter proper, which endows the osteoid layer with a considerable degree of vitality and sensibility.

The peculiar interzonal formations, represented in Fig. 5, we have also examined with higher powers of the microscope. (See Fig. 7.)

We notice small protoplasmic bodies branching and interconnecting. Their offshoots run upward into cement-corpuscles, or into piercing fibres of the cementum; whereas, downward the offshoots blend with the dentinal fibres. Even the dentine is possessed of protoplasmic formations, a certain distance away from the interzonal layer; all of which proves a deficient calcification just at the time of the beginning of the development of the cementum. The specimen, from which the drawing is made, was not stained with chloride of gold, and not decalcified; hence the reticulum of the basis-substance is only indicated, but not plain. No doubt, however, can arise about its presence.

### V. CEMENTUM OF THE NECK.

Bödecker, in his above-quoted essay, was the first to accurately describe the minute anatomical features of the cementum of the neck. How deficient the knowledge of this portion was, even sixteen years ago, is best shown by a quotation from Charles S. Tomes, who says,[1] "Where the cementum is very thin, as for instance where it commences at the neck of a human tooth, it is to all appearance structureless, and does not contain any lacunæ." The cementum of the neck, as is well known, forms an extremely thin layer, sharply bordered towards the enamel, and sometimes even overlaps the latter. Bödecker speaks of directly calcified prisms which, in his opinion, may be considered as osteoblasts, infiltrated with lime-salts. Sometimes the prismatic structure is not pronounced, but a row of spindles is seen arranged in an almost vertical direction to the surface of the tooth. Larger and smaller spindles alternate, the latter sometimes being so small as to convey the impression of fibres.

There is a slight discrepancy between the statements of Bödecker and our own observations. We fully concur with him as to the calcified prisms; but cannot consider the spindles, sometimes very conspicuous even to medium powers of the microscope, as formations of the basis-substance. We would consider the spindles and alternating fibres as protoplasmic formations, which fill the interstices between the prismatic pieces of the cementum of the neck. This tissue, in our opinion, is either composed of prisms alone, with intervening light and parallel spaces, or of prisms, between which are protoplasmic bodies of spindle shape, alternately broad and narrow, in the latter instance having the aspect of coarse fibres of living matter. Thus the uniformity in the structure of the cementum of the neck is established. We hope Dr. Bödecker, with whom we thoroughly concur in all other points, will pardon our heresy and eventually satisfy himself that our conception renders this structure more comprehensive than his. (See Fig. 8.)

Our view finds support in a drawing of Bödecker's above-quoted great essay, entitled "Anomalous Formation of Cementum of the Neck of a Human Tooth." In this figure dentinal fibres are seen bifurcating, and here and there inosculating with the spindles between the prisms. Such a connection is possible only if the spindles are protoplasmic formations.

Considerable interest attaches to the layer of dentine directly subjacent to the prismatic layer of the cementum of the neck. We are indebted to John Tomes for the knowledge of the fact that the dentinal fibres, in this situation, stop short of the cementum.

---

[1] "A Manual of Dental Anatomy," Philadelphia, 1876.

We can say that there is no region in the structure of the teeth varying in aspect so greatly as the dentine in the region of the neck. There are scarcely two teeth perfectly alike in this portion. The simplest form is when the prismatic layer is directly followed by a granular layer of dentine, lacking canaliculi. That the granulation means a rich reticulum of living matter, coarser than in any other part of the dentine, and rendering the neck so excessively sensitive, John I. Hart has proven, fully in accord with Bödecker's statements. This simple arrangement, however, is rare. In many instances there are layers of stratifications between the cementum and the granular dentine, again greatly varying in breadth and in numbers. The appearance of strata, in accord with the conception of John I. Hart, means that alternate layers, rich in living matter, and others scantily supplied with it, build up the neck-region of the dentine. Club-like or pear-shaped spaces, filled with protoplasm, are often seen to pervade the granular layer of the dentine with a peculiar regularity, often in connection with dentinal fibres, which otherwise do not reach the boundary of the cementum. In Fig. 8 we have represented coarse, dark-violet dots in the granular layer of the dentine, forming points of intersection, and far too small to be termed protoplasmic bodies or cells in the sense of older histologists. All these features seem to mean a markedly augmented amount of living matter, which renders all surgical interference in the region of the neck so painful.

The most complicated structure that we have ever seen in the region of the neck is illustrated in Fig. 9.

The figure is taken from a bicuspid, showing at its crown a distinct mechanical abrasion, ground by Dr. Bödecker. The stratifications extend all over the root. It is impossible to bring them into a relation with the mechanical abrasion, and we would consider this formation a rather anomalous one. The layers are as follows: The outermost portion, directly bordering the cementum, is composed of large, regular prisms, which show faint longitudinal lines and are halved by distinct fissures in a direction parallel to the surface; this layer extends to the close vicinity of the enamel, and far down along the root, directly changing into stratified cementum, devoid of cement-corpuscles, as far as the specimen shows. This is the only layer that belongs to the cementum, since in the neighborhood of the enamel the dentinal fibres are traceable up to the prisms, pervading a granular basis-substance, as yet but slightly stratified. Soon afterwards the dentine begins to exhibit a number of layers, which may be defined as a narrow outer, and a broad inner, lamellated stratum, between which lies an outer granular zone, somewhat varying in breadth along the root. These three layers are pierced by extremely delicate radiating lines, which closely resemble dentinal fibres. The next layer is the inner granular, greatly changing in breadth in different portions of the root. This layer holds small, angular protoplasmic formations, and, obviously, corresponds to the granular layer subjacent to the cementum of the neck in normal teeth. Inwardly there follows a zone (D–1) marked by a high gloss, due to an intense calcification of the basis-substance, pierced by scanty angular protoplasmic bodies. The innermost stratum appears to be composed of prisms or spindles, far less regular than those of the cementum. A sharp line of demarcation closes this layer towards the normal dentine, in which not the slightest anomaly can be made out. All these layers are seen down to nearly half the length of the root, where the specimen is cut off.

Stratification is exceptionally observed in the dentine of the roots, consisting of narrow, light lines, arranged parallel to the surface, causing an interruption in the course of the dentinal fibres, but never a change in their direction. Stratification of the dentine at the neck is far more common, but was never seen as yet to such a perfection as in the specimen illustrated in Fig. 9. What the cause of such stratification is, future studies in the history of development of the dentine of the roots and the cementum will elucidate.

### VI. DEVITALIZED CEMENTUM.

We have ground a number of teeth which, judging from their naked-eye appearance, were pulpless and devitalized. After exposure of thin slabs obtained from such teeth to the solution of chloride of gold, with subsequent decalcification with acetic acid, the image under the microscope was striking. (See Fig. 10.)

The most conspicuous features were the empty lacunæ, destitute of nucleated protoplasm, but containing granular clusters of a dark-violet color, obviously the shrivelled remains of protoplasm. Numerous canaliculi arose from the lacunæ, all of which were empty. The finest offshoots of both the lacunæ and canaliculi produced a light net-work in the dark-violet basis-substance, an image which corresponds to dry and also to necrotic bone, as first described by Dr. Bödecker. Unfortunately, some specimens of living, freshly-extracted teeth furnished, after the same chemical treatment, identical figures. As mentioned in the chapter on "Methods," such an untoward result has also occurred to other investigators who resorted to the gold method. The cause of such a failure may be that the extracted teeth were left too long under the influence of the table-salt solution ; or, possibly, the acetic acid worked in a wrong way. All we can say is, that whenever such a negative result was obtained, it had affected the whole cementum, so much so that we are at a loss to state whether or not the devitalized cementum is dead all through up to the pericementum. Future examinations, carried out with improved methods, will probably settle the question where the boundary between dead and living tissue is, this being a matter of the greatest importance to the practitioner.

## Figures and Tables

**Fig. 1. f1:**
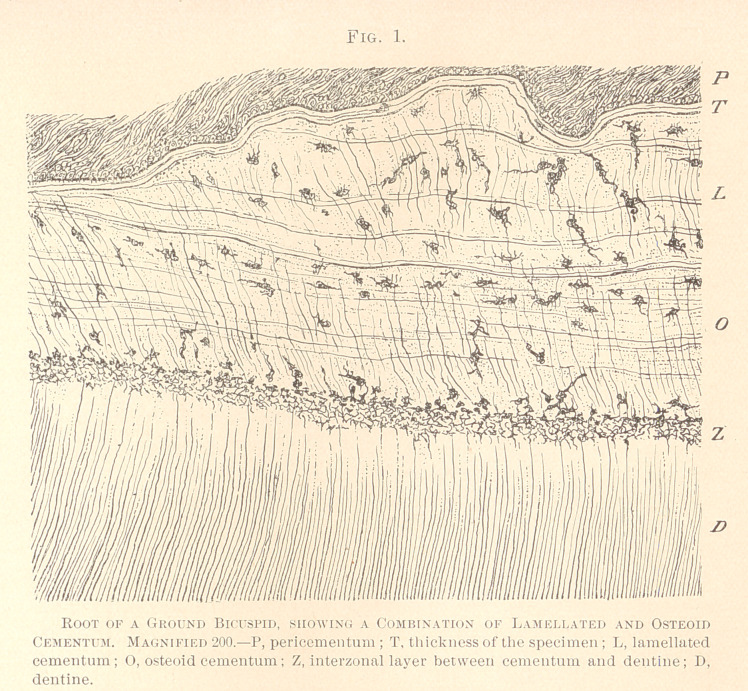


**Fig. 2. f2:**
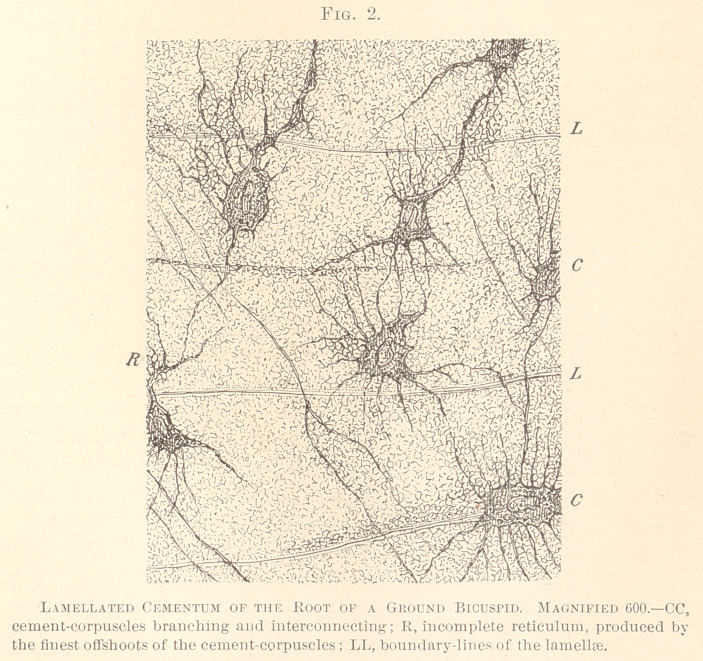


**Fig. 3 f3:**
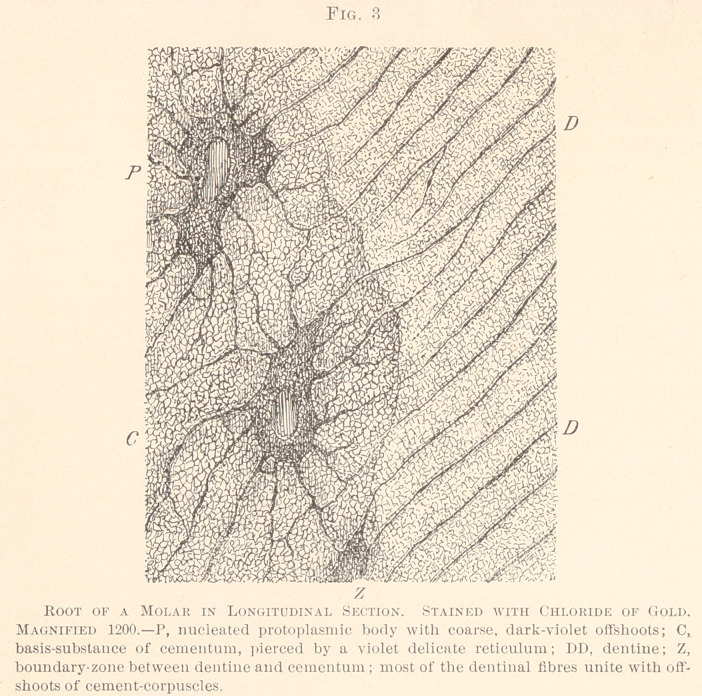


**Fig. 4. f4:**
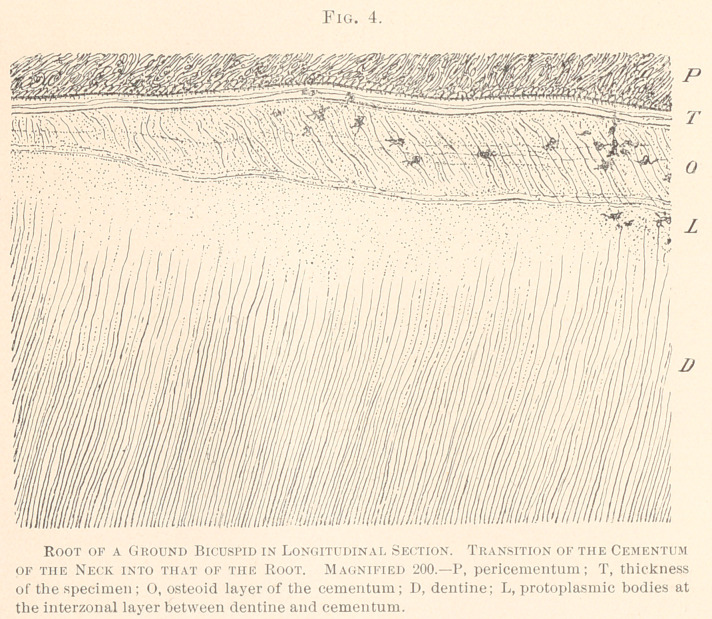


**Fig. 5 f5:**
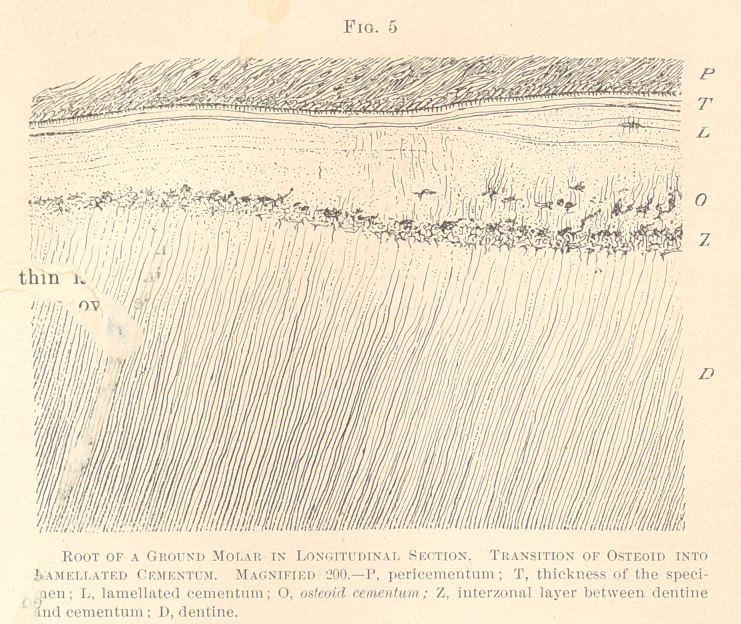


**Fig. 6. f6:**
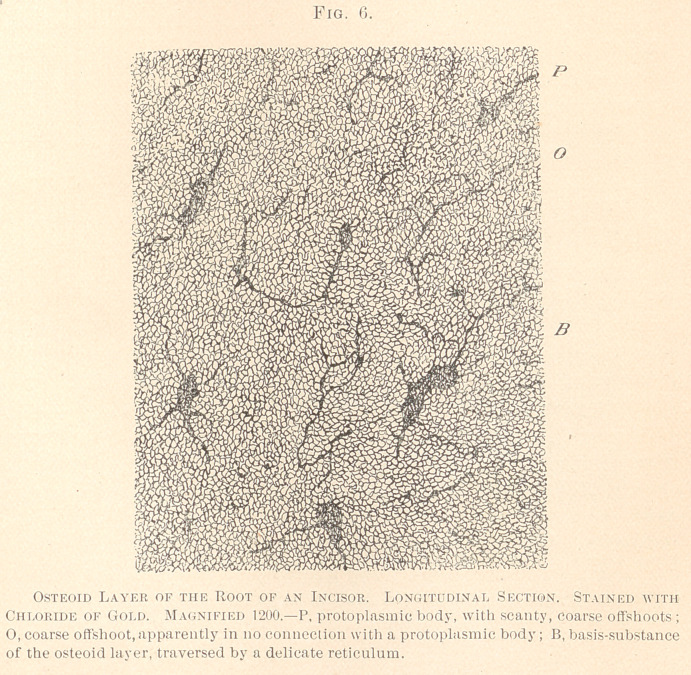


**Fig. 7. f7:**
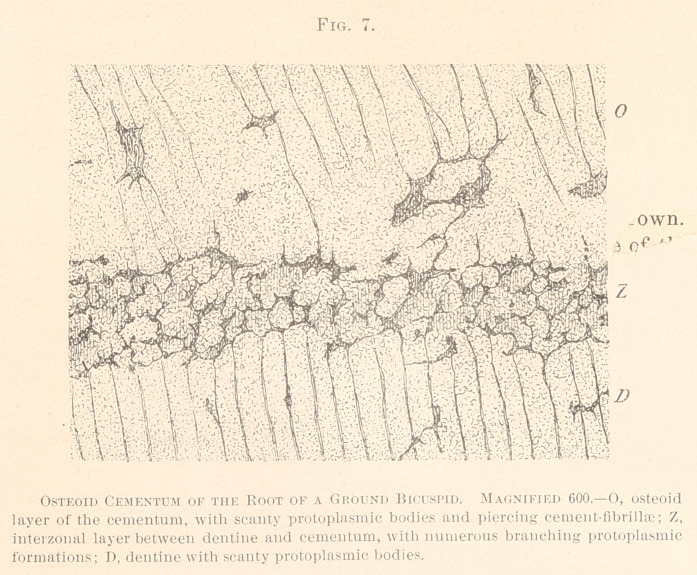


**Fig. 8. f8:**
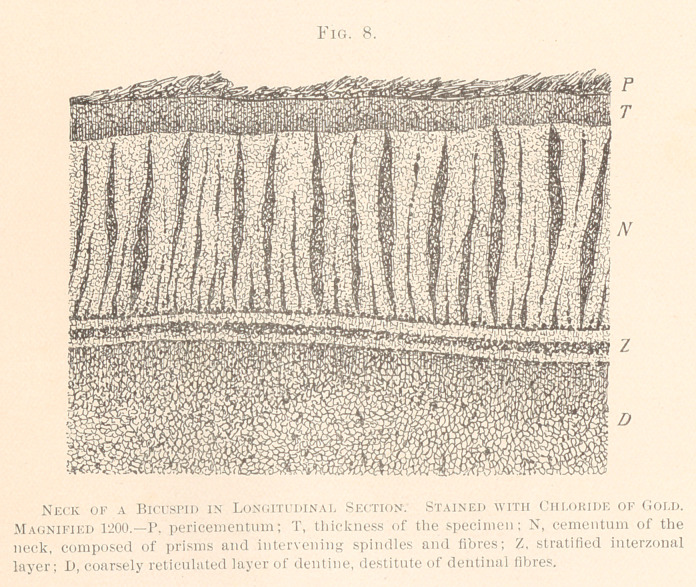


**Fig. 9. f9:**
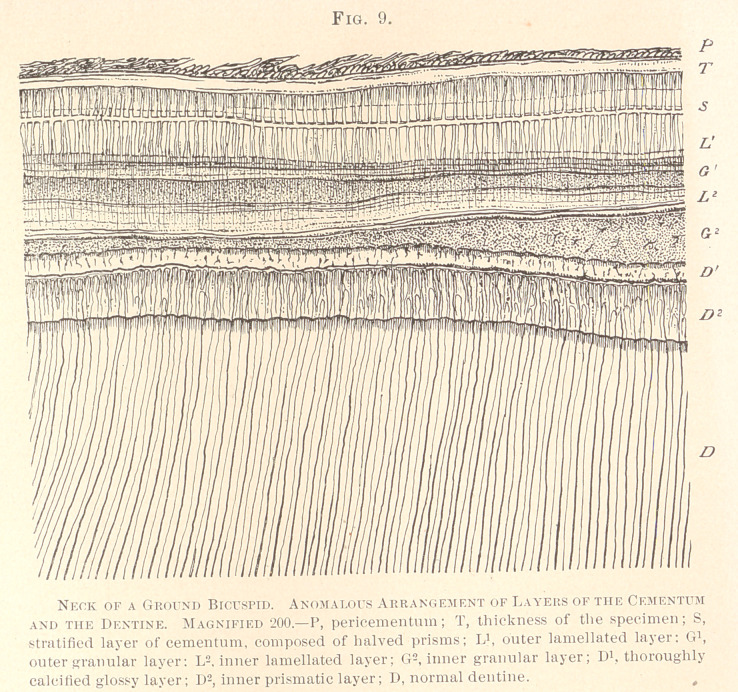


**Fig. 10. f10:**